# Palliative care conversations with people with dementia who live alone: untapped dimensions from a lived experience

**DOI:** 10.3389/frdem.2026.1791608

**Published:** 2026-04-15

**Authors:** Lesley E. Williamson, Dawn Horne, Rasa Mikelyte, Elisabeth B. Grey, Pippa Collins, Christopher Poyner, Annabel Farnood, Tomasina M. Oh

**Affiliations:** 1 NIHR Applied Research Collaboration South London; Cicely Saunders Institute of Palliative Care, Policy & Rehabilitation, and NIHR Health & Social Care Workforce Research Unit, The Policy Institute, King’s College London, London, United Kingdom; 2 Person Living with Dementia, Canterbury, United Kingdom; 3 NIHR Applied Research Collaboration Kent, Surrey and Sussex; Centre for Health Services Studies, University of Kent, Canterbury, United Kingdom; 4 NIHR Applied Research Collaboration West; Population Health Sciences, Bristol Medical School, University of Bristol, Bristol, United Kingdom; 5 NIHR Applied Research Collaboration Wessex; School of Health Science University Southampton, Southampton, United Kingdom; 6 Rural Mental Health Research Unit, Department of Psychology and Mental Health, School of Health and Wellbeing, University of Worcester, Worcester, United Kingdom; 7 NIHR Applied Research Collaboration South London; Cicely Saunders Institute of Palliative Care, Policy & Rehabilitation, King’s College London, London, United Kingdom; 8 NIHR Applied Research Collaboration South West Peninsula; Community and Primary Care Research Centre, Peninsula Medical School, Faculty of Health, University of Plymouth, Plymouth, United Kingdom

**Keywords:** advance care planning, clinical conversations, dementia, end-of-life care, living alone, palliative care, healthcare disparities

## Abstract

Dementia is a life-limiting condition, and a palliative care approach can improve both quality of life and quality of care for people living—and dying—with dementia. Research has consistently shown that, compared with other terminal conditions such as cancer, dementia is under-recognised and under-treated within palliative care systems. Considerable attention has been paid to this overall under-representation; however, further inequities exist within palliative dementia care research and practice itself. This position paper draws on an in-depth conversation with a person living alone with Alzheimer’s disease and vascular dementia, alongside a critical engagement with existing literature. Using lived experience as a starting point, we identify two untapped dimensions of palliative dementia care: (1) barriers in palliative care conversations when a person with dementia attends clinical appointments unaccompanied; and (2) intersectional disadvantage arising from dementia, living alone, and health and social care systems that overly rely on informal carers or supporters. These contribute to exclusionary research practices that marginalise people living with dementia without close care partners. While the involvement of carers and supporters in shared decision-making should be encouraged when they are present, high-quality palliative care and research must not depend on their presence and should be equally accessible to people living with dementia who attend services alone. We argue that addressing this neglected area requires the meaningful involvement of people with lived experience in shaping both research agendas and clinical practice.

## Background

The provision of a palliative care approach for people with dementia can promote quality of life (den Van Block, 2014) and end-of-life care (Dixon et al., 2018; Williamson et al., 2021). However, palliative care needs of people with dementia are often underassessed and undermanaged in practice (Dempsey et al., 2015; Leniz et al., 2021). The number of people with dementia and palliative care needs is projected to increase substantially by 2040 (Yorganci et al., 2024), as is the number of people with dementia living alone (Gamble et al., 2025) who also report feeling ‘invisible’ (Frazer et al., 2012). Already 40% of people with dementia live alone (Gamble et al., 2025), thus research to understand how care contacts can best serve people with dementia presenting alone will become increasingly important. There is strong evidence on the role of the carer or supporter in palliative care provision (Reigada et al., 2015), and a growing evidence base on the unique care needs experienced by people living and dying with dementia who live alone (Crance and Yu, 2025). However, there is little evidence on support for palliative and end-of-life care for people living alone with dementia (Polack et al., 2025).

Dementia research has historically centred on people with dementia who live with carers, often overlooking those who live alone (Crance and Yu, 2025; Polack et al., 2025; Cheung-Cook et al., 2025). The same pattern appears in studies on advance care planning (ACP)—one of the key elements of a palliative care approach in the care of people living with dementia. A systematic review by Sellars et al. (2019) found that carer perspectives are far more prominent than those of people living with dementia in ACP research. When people living with dementia are included, it is most often through person with dementia–carer dyads rather than as independent participants (Liu et al., 2024; Hansen et al., 2020). Many interventions and studies require a cohabiting care partner, family supporter or proxy, especially as cognitive decline progresses (Liu et al., 2024; Jones et al., 2019); in research with those at later stages of dementia it is common to see the carer involved on their own (Brazil et al., 2018). The upshot of this is that people living alone with dementia are less likely to be recruited. Although some people with dementia who live alone may still participate alongside a non-resident supporter (e.g., an adult child who lives elsewhere), the field’s reliance on family and friend carers strongly suggests a systematic underrepresentation of this group. Unclear also is whether outcomes from ACP discussions for people living alone or who attend these discussions without their supporters are different from those where a carer is present.

The lack of representation of people living alone with dementia in palliative care research is likely to be mirrored when it comes to care planning in healthcare practice. Practice is informed by evidence from patient-carer dyads or patient-carer-professional triads (Bybee et al., 2025; Yeung et al., 2023). As a result, best-practice evidence tends to uphold practice models that assume supporter involvement, offering little guidance for practitioners supporting people living with dementia who undertake ACP independently (National Institute for Health and Care Excellence (NICE), 2018). This cycle increases the risk that high-quality practice favours those where there is carer involvement.

This observation is not meant to dissuade practitioners from taking a relational approach to ACP in dementia—we acknowledge that people living with dementia co-construct their needs together with care partners/family members and often make decisions interdependently (Phenwan et al., 2025)—this approach is both needed and welcome for those with close supporter involvement. Moreover, the inclusion of supporters in ACP and palliative care discussions as well as shared decision making in healthcare consultations should be encouraged and is emphasised in clinical recommendations (National Institute for Health and Care Excellence (NICE), 2018; Piers et al., 2018). Evidence shows that ACP completion is more likely with multiple conversations over time (Kelly et al., 2019); some people with dementia’s need for support to reflect on their values and think about their future care (Piers et al., 2018)—a role often fulfilled by a family carer—may therefore increase over time. However, where this is not possible or not the wish of the person with dementia (both of which may be more likely for people living alone), the clinician may need to adopt a different approach, such as providing extra support.

To truly understand the nature and extent of these untapped aspects of dementia research and practice—namely ACP-related conversations with people living alone with dementia and attending appointments without a supporter or care partner—it is crucial to start from a position of lived experience. For this reason, the team behind this position paper comprises not only applied dementia care researchers from across England, but also Dawn Horne who lives alone with Alzheimer’s disease and vascular dementia. Together, we had an in-depth, semi-structured discussion about palliative and end-of-life care for people with dementia. Dawn’s personal experiences, captured as part of this conversation, exemplified the challenges experienced by both the person living with dementia and care professionals to negotiate palliative care discussions in the absence of a care partner, highlighting important gaps in dementia research. Some sections of the conversation have been published elsewhere (Horne et al., 2026). Here we present excerpts of Dawn’s experiences to illustrate these untapped dimensions. These excerpts are accompanied by associated literature and researcher commentary.

### Untapped dimension 1: communication barriers

The personal story Dawn has shared in Box 1 above underscores the difficulty of someone living with dementia advocating for their advance care planning wishes while on their own, without a care partner present. The impact of care partners in the clinical consultation is well known. While there are observations of some care partners dominating consultations (Sinclair et al., 2021), they can play a crucial informant role to complement a comprehensive clinical assessment (Dooley et al., 2015). This becomes increasingly important for people with advancing dementia whose condition makes decision-making and articulating care preferences difficult for others to comprehend, resulting in an increased reliance on the support of those around them (Lindeza et al., 2020; Sinclair et al., 2019). Care partners can also play a key advocacy role (Dooley et al., 2015; Sussman et al., 2021), championing the rights of those affected by dementia and providing ‘candidacy-by-proxy’ (Williamson et al., 2023). This is especially important in the context of diagnostic overshadowing and failure to prioritise dementia as a life-limiting condition (Williamson et al., 2023; Pepper and Dening, 2024).

BOX 1 **Transcribed extract from a conversation with Dawn Horne**

They asked me whether I had a do not resuscitate order, and I said that I did not. She told me I needed to talk to a doctor, so I went to a doctor.

I asked him whether I could put a do not resuscitate plan in place, and he refused. He said, “You’re doing okay now - you do not need it.” I told him that I knew I was doing okay, but that I needed to think ahead. He said he would think about it and get back to me, and that we would make an appointment. But he never did.

So it never happened.

From my past experience working as a psychiatric nurse, I’ve seen what happens when people have not signed one - being resuscitated when life was already awful, when they did not want to come back. I do not want to be in that position.

He [the doctor] clearly wasn’t prepared to talk to me about it. That was especially surprising because he’s the doctor I see most often, and the one I try to see whenever I can. He has the most experience with people with dementia and the most understanding. Even so, he did not want to be involved in signing a do not resuscitate form for me. I think he simply did not want to take the responsibility.

It’s left me up in the air [.] left me with the worry that in the future I might be resuscitated when I do not want to be. It’s also put pressure on my family, and I’m trying to make decisions now so that they do not have to later. [.]

I would like to have somebody I could lean on, so we could look at the options together and make a decision. [.] I would not want someone to do it for me, but I would hope there was somebody I could lean on enough to support me through it. I think that’s probably what most people would want, even if it is not always possible.

The role of care partners as informants and advocates underscores the relational view of disability in dementia, which considers the functional barriers imposed by the disease and sociocultural barriers imposed by society (Shakespeare et al., 2019). With this lens, lone contacts with care services pose communication barriers for people with dementia, either because of their condition or how it is viewed, or both. This may account for why some people with dementia can feel vulnerable when attending services alone, particularly in acute care settings (Williamson et al., 2023). Such vulnerability is likely to further compound discussions around future care planning, which are already limited by challenges around initiating these with professionals (Sellars et al., 2019).

Lone contacts by people with dementia may also pose communication challenges for health and care professionals, who typically welcome the involvement of care partners in decision-making (Sinclair et al., 2021). The absence of care partners may thus challenge the confidence of practitioners to discuss care decisions, exacerbating the complexity of balancing autonomy against risk (de Witt and Ploeg, 2016; Waugh, 2009). It may also challenge normative beliefs about people with dementia, who are often viewed as a homogenous group of patients with similar living situations (Odzakovic et al., 2021). However, people with dementia who live alone can have strong neighbourhood connections and contacts with formal carers (Odzakovic et al., 2021). Therefore, consultations with people with dementia presenting alone may require less focus on involving the ‘next of kin’ and more on integrating care with an individual’s personal circumstance and social network.

In general, research looking at communication and consultations often involves the family as well as the person with dementia. There are fewer studies conceptualising communication and service interaction among people with dementia without a care partner present. However, the potential barriers to communication highlighted by the reflection above are an important but presently untapped dimension of dementia research. Understanding these barriers will inform efforts to empower people with dementia and practitioners to fully discuss care needs and wishes, including those towards the end of life.

### Untapped dimension 2: intersectional disadvantage

Dementia is a known source of social disadvantage (Biggs et al., 2019); in care settings, this can manifest as a ‘culture of dementia care’, in which the needs and preferences of people with dementia are overlooked (Martin et al., 2020). This is illustrated in Box 2, Extract 1, reiterating evidence of people affected by dementia needing to push and fight for care (Williamson et al., 2023; Prorok et al., 2017). Research on illness identity suggests that access to healthcare is influenced by the sociocultural representation of illnesses, where conditions, like cancer, are of greater social significance and systemic sympathy compared to other conditions (Macdonald et al., 2016). With ageism and mental illness stigma surrounding dementia (Milne, 2010), it not only falls short of systemic sympathy but may also be subject to systemic apathy (Williamson et al., 2023). While this lack of parity of esteem between other life-limiting conditions is not new, its impact continues to drive inequity in accessing palliative care (Davies et al., 2014), advance care planning (Hommel et al., 2025), and high-quality end-of-life care (Martinsson et al., 2018).

BOX 2 **Transcribed extract from a conversation with Dawn Horne**

**Extract 1**: At the doctor’s you see a different doctor almost every time. On this occasion I saw someone I’d never met before. I needed some painkillers, and at some point I said to him, “Of course, it’s a bit difficult, because I have Alzheimer’s.” He then seemed to read my notes, and after that he completely switched off.

By the end of the appointment, I just said, “Are you going to give me pain killers?” and he said, “I suppose so.” It’s the only time I’ve ever experienced that with a professional, but it was very noticeable. Later, I spoke to another doctor about it and said, “Please do not let me see him again.”

Most of the time, if I’m in trouble when I’m out - if I’m in the bank and I cannot do something - I tell people there and ask for help. I say I have Alzheimer’s, because people recognise it. I do not say dementia. People have always been helpful.

I did not expect that reaction from a doctor.

**Extract 2**: Everything I’ve done to help with my Alzheimer’s, I’ve done through my own research and my own experience. Nobody has told me what I could do, what I could not do, or even what my options were.

When I was diagnosed, COVID was happening. There were no living with dementia groups or anything like that. It was simply a diagnosis - a dementia diagnosis. Nobody ever talked things through with me or explained what it meant. [.] Even though we are supposed to have an ECG every six months, that does not happen. It just does not happen. You’re on your own.

In my experience, I hear other people talk about having follow-ups and support in place, but that has not happened for me, and nobody has told me what to expect. I’ve had to find out the hard way. That’s okay for me, but it cannot be okay for everybody. There must be other people who are completely lost - people who stay at home and get worse because nobody has told them how not to get worse.

Dementia intersects with other social identities (Aspinal et al., 2023), such as those pertaining to gender, age, and ethnicity, which may drive complex discrimination in health and social care. Not having a care partner may represent another intersecting social identity that disadvantages people with dementia in the health and social care system. Care partners are considered fundamental to the provision of best practice dementia care (Worthington et al., 2023), and with systemic dependency on them to access, coordinate and provide care to people with dementia (Henderson et al., 2022), their absence leaves a significant service gap. As Box 2, Extract 2 illustrates, those without the capital needed to develop dementia literacy may experience complex disadvantage without a care partner, particularly in a system of variable availability of post-diagnostic support (Frost et al., 2021). Based on previous literature, care partner absence may also have an impact on care access and quality due to, for example, lack of transport for appointments (Polack et al., 2025) or advocacy of end-of-life care preferences (Poole et al., 2018).

Given the existing risk of intersectional discrimination of people with dementia without direct care partners, it is essential that we research how we can best support palliative care and end-of-life care for people with dementia who have no care partners or choose to attend alone. An intersectional lens will help to explore the nuance of dementia inequality and inform efforts to personalise care (Cheung-Cook et al., 2025).

### Concluding reflections

We hope this short piece has highlighted how dementia research often privileges the voices of people with dementia who live with care partners (Crance and Yu, 2025; Polack et al., 2025), including research into palliative dementia care (Liu et al., 2024; Hansen et al., 2020). From this apparent selection bias, we know comparatively little of the needs of people with dementia presenting to services alone and if their needs are being met. Our in-depth conversation and the points highlighted above would appear to support this, underlining the need for research that is inclusive not only of those who have the support of care partners but *also* of people who live or present to services alone.

Research excluding people with dementia who present to services alone may be intentional, aiming to specifically explore the dynamics of dyad relationships or only the views of care partners. Or it may be inadvertent, due to normative assumptions of people with dementia. Research ethics processes, especially those around personal consultee assent when a person living with dementia is deemed to lack capacity for informed consent to take part in a particular research study, can also result in exclusion (de Meiros et al., 2022). The current dearth of research in this area may also reflect the challenges of recruiting people with dementia who live or attend appointments alone (Aspinal et al., 2023), requiring purposive recruitment strategies that are co-designed and draw on established partnerships with community providers serving marginalised groups (Assfaw et al., 2025). A co-productive approach may also ensure dementia research is inclusive, equitable, and genuine for those without direct care partners, with the potential to inform policy and practice (de Sans-Guimarães et al., 2022). This approach ensures people with dementia are in the ‘driving seat’ of research (Litherland and Hare, 2024), with shared vision, responsibility and symbolic capital (Mitchell et al., 2024). It thus provides opportunity for meaningful and just involvement of people with dementia who live or present to services alone.

Overall, people living alone with dementia can feel ‘invisible’ in society (Frazer et al., 2012) and are seemingly at risk of being invisible in both the consultation room and research paper. However, receiving good quality palliative and end-of-life care should not be determined by the presence or absence of a care partner. As more people with dementia will be living and interacting with services alone, we must spotlight this marginalised population by researching the untapped dimensions of communication and intersectional disadvantage in palliative and end-of-life care. As dementia researchers, we must also ensure our research is purposively inclusive of people who present to services alone, promoting their visibility in the system and within society—not only as participants, but as co-researchers guiding the research process from the idea stage to delivery.

## Data Availability

The original contributions presented in the study, including the personal and identifiable statements from co-author Mrs Dawn Horne, are included in the article/supplementary material; further inquiries can be directed to the corresponding author.
